# Influence of single shot and continuous peripheral nerve block on opioid consumption and perioperative pain management in patients undergoing total elbow arthroplasty

**DOI:** 10.1016/j.jseint.2025.03.003

**Published:** 2025-03-28

**Authors:** Tamara Babasiz, Michael Hackl, Sebastian Wegmann, Jan Hockmann, Kai Hoffeld, Lars P. Müller, Tim Leschinger

**Affiliations:** Faculty of Medicine and University Hospital Cologne, Department of Orthopaedic and Trauma Surgery, University of Cologne, Cologne, Germany

**Keywords:** Total elbow arthroplasty, Elbow osteoarthritis, Peripheral nerve block, Nerve block catheter, Regional anesthesia, Perioperative pain management, Opioids, Retrospective study

## Abstract

**Background:**

Peripheral nerve block (PNB) is effective for pain management after shoulder arthroplasty. Our study investigated the impact of regional anesthesia on perioperative pain management following total elbow arthroplasty (TEA). We aimed to determine whether single shot anesthesia (SSA) or continuous peripheral nerve block (CPNB) reduces opioid usage and improves postoperative pain levels compared to general anesthesia alone.

**Methods:**

A retrospective analysis evaluated 78 patients who underwent TEA for perioperative pain management, including nonopioid, opioid medications, and on-demand opioid use. Postoperative pain was assessed using the Numeric Rating Scale (NRS). Patients were divided into 3 groups based on anesthesia type: Group 1 received a PNB intraoperatively and a CPNB; Group 2 received SSA; and Group 3 underwent general anesthesia alone. Pain management and perception were compared over 5 days postoperatively using the Mann-Whitney U test.

**Results:**

Group 1 had significantly lower intraoperative opioid usage compared to group 3 (*P* = .0423) and required fewer opioids postoperatively (*P* = .0114). Group 1 also reported lower NRS scores, indicating better pain relief (*P* < .0001). Group 2 showed a trend toward reduced intraoperative (*P* = .4372) and postoperative opioid (*P* = .1107) use compared to group 3, although with no statistical significance. Group 2 had significantly lower NRS scores than group 3 (*P* = .0023).

**Conclusion:**

In this first study on PNB and CPNB in TEA, we showed that CPNB reduces perioperative opioid use and improves postoperative pain. SSA offers no significant advantage over CPNB in reducing opioid usage. Avoiding systemic opioids and their adverse effects is particularly beneficial for elderly patients.

Any type of surgery may result in persistent postoperative pain that can reduce quality of life, cause functional impairments, and lead to patient distress.^22^ Chronic postoperative pain remains a widespread issue, affecting approximately 20% to 30% of patients worldwide. Effective pain prevention requires appropriate anesthetic support and effective management of severe acute pain following surgery.^3^

Focusing on surgical interventions for elbow injuries, total elbow arthroplasty (TEA) has become increasingly common, particularly in cases arising from acute trauma or post-traumatic conditions.^13^ TEA frequently leads to intense postoperative pain, often necessitating hospitalization for effective pain management.^18^ The effective control of pain during hospitalization is a crucial factor influencing both postoperative outcomes and patients’ subjective experiences following TEA.

The use of peripheral nerve blocks (PNBs) during lower extremity surgery has been well established in endoprosthetic procedures for decades.^14^ Numerous studies have shown that PNBs in joint arthroplasty of the lower extremity provide pain relief and superior functional outcomes compared to systematic analgesia, while also exhibiting fewer side effects.^15^ Moreover, continuous peripheral nerve block (CPNB) catheters are frequently used postoperatively for pain management following joint replacements, particularly of the knee and hip.^4^ PNBs and additional catheters, employed in surgical settings, have been associated with several advantages, including enhanced pain relief, reduced reliance on narcotics and perioperative opioid consumption, better sleep quality, and most notably increased patient satisfaction.^8^ In this context, it is important to highlight the need to mitigate opioid-related side effects by reducing opioid consumption, such as gastrointestinal issues (nausea, vomiting, and ileus), cognitive effects (somnolence, dizziness, and delirium), pruritus, urinary retention, and respiratory depression. These adverse effects of systemic opioid usage are particularly concerning in elderly individuals and may have long-lasting consequences on overall patient outcomes, independence, and quality of life.^15^

Focusing on the upper extremity, studies for shoulder surgery have demonstrated that the use of a regional nerve block significantly reduces the need for patient-controlled analgesia postoperatively.^7^ Additionally, a study on the use of an interscalene nerve block for pain management demonstrated effective reduction of pain and opioid consumption after total shoulder arthroplasty.^13^ Therefore, a PNB is presumably an effective pain management option after shoulder arthroplasty with increasing popularity over the past decade.^6^ Data on the use of regional anesthesia, single shot anesthesia (SSA), and postoperative nerve block catheters in TEA are lacking, especially regarding their impact on opioid utilization and patient-reported pain perception. Therefore, in this study, we examined the influence of SSA and CPNB following elbow arthroplasty, with a focus on perioperative pain medication consumption and subjective pain levels. Our hypothesis was that patients receiving additional SSA or nerve block catheters require less opioids and exhibit better postoperative pain levels.

## Materials and methods

The retrospective study was approved by the Ethics Commission of Cologne University's Faculty of Medicine (registration number: 22-1278). A single-center study was performed on patients who underwent TEA between May 2021 and November 2023 at the University Hospital of Cologne (Cologne, Germany). The surgical indications encompassed nonreconstructable acute fractures, post-traumatic osteoarthritis, and primary osteoarthritis. Patients were included if TEA was performed for any of the aforementioned specified indications and complete documentation during the follow-up period was available. Patients who received additional surgical procedures were excluded, as were patients who did not complete the postoperative evaluation forms during the postoperative period. Upon application of the specified criteria, a total of 78 electronic patient files were assessed.

Group 1 included 45 patients, which underwent TEA under general anesthesia (GA) with additional PNB intraoperatively combined with a nerve block catheter for pain therapy after surgery (CPNB). Group 2 included 19 patients, which underwent surgery for TEA under GA with additional temporarily regional anesthesia in terms of SSA intraoperatively, but without nerve block catheter postoperatively. Group 3 included 14 patients, which underwent TEA only under GA without additional SSA or CPNB.

Before surgery, we recorded patients’ sex, age, dominant limb, and history of previous operations. In addition, we analyzed intraoperative opioid usage as well as postoperative usage of nonopioid pain medication (ibuprofen and metamizole) and postoperative opioid usage (eg, Morphine, Tilidine, Oxycodone) and postoperative on-demand opioid pain medication. To standardize opioid dosages to morphine equivalents, opioid dosages were converted using the appropriate conversion factor (0.2 for Tilidine and Tramadole; 2 for Oxycodone). Total dosages of each pain medication (ibuprofen, metamizole, and opioids) administered during the first 5 days postoperatively were added up for each patient. Mean and standard deviation of each group were calculated and analyzed.

Importantly, all patients included in this study were treated in an inpatient setting. Postoperative pain medication and all other medication were systematically recorded in each patient’s digital medical record using the Meona patient record software (Meona GmbH, Freiburg, Germany). All patients received the same pain medication scheme, including predefined opioid and nonopioid pain management strategies based on World Health Organization guidelines, if not contraindicated. Intraoperative analgesia was administered using the opioid sufentanil (μg). Additionally, intraoperative opioid administration was documented using the anesthetists' paper records, which were subsequently stored in the hospital's electronic medical system.

The PNB was performed under ultrasound guidance, targeting either the interscalene, supraclavicular, or axillary nerves, depending on individual patient conditions and the attending anesthesiologist’s decision. Intraoperative SSA were applicated using 7.5-10 mL 0.5% or 1% ropivacaine and 7.5-10 mL 1% or 2% prilocaine, depending on individual patient conditions. Postoperative CPNB consisted of 0.2% ropivacaine, which was administered continuously with a rate of 4-12 mL per hour, based on the individual patient's pain sensitivity and tolerance, as well as their weight and height. The catheter was placed during the induction of anesthesia and remained in place postoperatively, without being replaced. Anesthesia was administered through the preplaced catheter. Patients who received continuous nerve blocks were not restricted in movement and were able to fully participate in the postoperative physiotherapy-guided mobilization program. All catheters were overseen and rounded by a specialist anesthetist daily during hospitalization. The catheters were removed 24 hours before the patient was discharged, while patients were regularly seen before leaving the hospital once more by an anesthetist.

The surgical techniques of TEA were recently described.^13^ After surgical intervention, all patients underwent a standardized clinical examination, which included daily assessment of pain using the Numeric Rating Scale (NRS) and pain therapy of clinical house standard depending on individual pain level. The NRS is a widely used tool for assessing pain intensity. It typically involves asking patients to rate their pain on a scale from 0 to 10, where 0 represents “no pain” and 10 represents “the worst possible pain.” NRS scores during the first 5 days postoperatively were added up for each patient. Mean and standard deviation of each group were calculated and analyzed.

### Statistical analysis

Statistical analysis was performed using GraphPad Prism 10 (GraphPad, San Diego, CA, USA). Distribution of the clinical outcome parameters was presented by the mean, standard deviation, and minimum and maximum values. We applied the Kolmogorov-Smirnov test to statistically evaluate normal distribution of sample groups. As we could not confirm normal distribution, we applied the Mann-Whitney U test to analyze differences between both groups. Chi-square test with Fisher’s exact test was performed to assess significance of distribution between groups. The alpha level was set to 0.05.

## Results

A total of 78 patients (15 females and 63 males) treated with TEA were included in this study and the participants were divided into 3 cohorts. Group 1 was made up of 45 patients and consisted of 9 women and 36 men. Group 2 comprised 19 patients, of which 3 were women and 16 were men. Group 3 consisted of 3 women and 11 men (Table I). None of the patients included in this study experienced any complications related to the PNB catheter.

**Table I:** Patient demographics.

	Group 1	Group 2	Group 3
N	45	19	14
Age (yr)	71.16 (± 11.11)	70.26 (± 4.99)	73.21 (± 8.59)
Sex			
Women	9 (20%)	3 (15.79%)	3 (21.43%)
Men	36 (80%)	16 (84.21%)	11 (78.57%)
Cause of elbow arthroplasty			
Primary osteoarthritis	4 (8.89%)	2 (10.53%)	1 (7.14%)
Post-traumatic osteoarthritis	10 (22.22%)	5 (26.32%)	4 (28.57%)
Acute trauma	31 (68.89%)	12 (63.15%)	9 (64.29%)
Number of previous surgeries	1.6 (± 1.9)	1.2 (± 1.2)	2.1 (± 2.25)
Duration of surgery	2.45 (± 0.76)	2.51 (± 1.04)	2.25 (± 0.66)
Duration of hospitalization	8.62 (± 3.24)	7.95 (± 3.44)	8.14 (± 1.73)
Nerve block type			
Supraclavicular	31 (68.89%)	10 (52.63%)	
Interscalene	10 (22.22%)	8 (42.12%)	
Axillar	4 (8.89%)	1 (5.26%)	

Group 1: received PNB, combined with nerve block catheter postoperatively; Group 2: received SSA, intraoperatively; Group 3: underwent only general anesthesia. Values indicate mean (± standard deviation), or exact values (percentage of whole). *PNB*, peripheral nerve block; *SSA*, single shot anesthesia.

Patients in group 1 received a PNB and an additional nerve block catheter for postoperative pain therapy. The nerve block catheter was in place for an average of 3.49 (±1.86) days postoperatively in this group.

**Figure 1:**
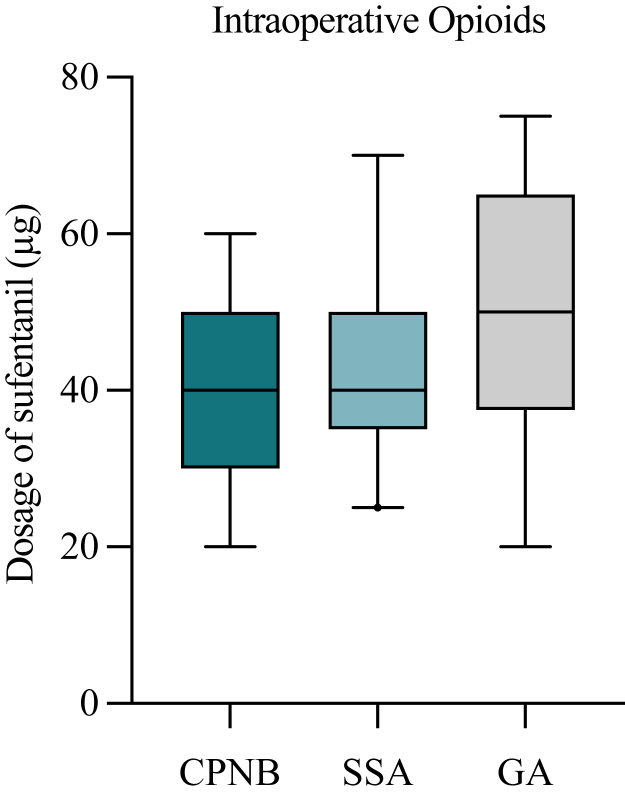
Intraoperative sufentanil usage. Total usage of sufentanil (μg) from induction of anesthesia until extubation is presented for each group. *CPNB*, continuous peripheral nerve block (group 1); *SSA*, single shot anesthesia (group 2); *GA*, general anesthesia (group 3). Values indicate median and 5-95 percentile range. Statistics were performed using Mann-Whitney U test.

Patients of group 1 required significantly lower dosages of intraoperative sufentanil, when compared with patients of group 3, which solely received GA (*P* = .0276) (Fig. 1).

**Figure 2:**
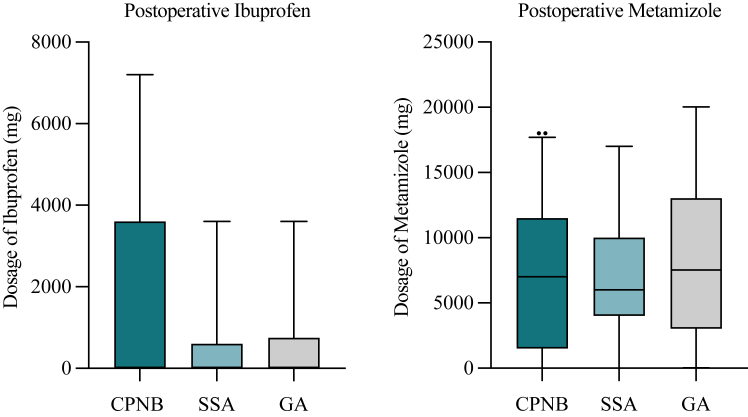
Postoperative pain medication (nonopioids). Total usage of ibuprofen (mg) and metamizole (mg) during the first 5 postoperative days for each group. *CPNB*, continuous peripheral nerve block (group 1); *SSA*, single shot anesthesia (group 2); *GA*, general anesthesia (group 3). Values indicate median and 5-95 percentile range. Statistics were performed using Mann-Whitney U test.

**Figure 3:**
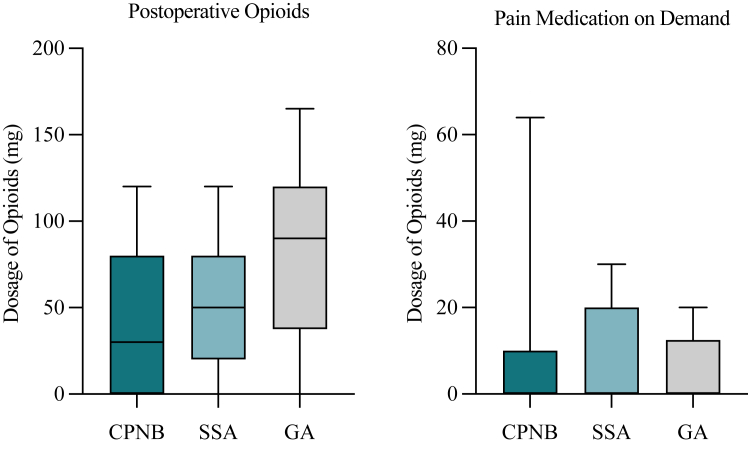
Postoperative pain medication (opioids). Total usage of opioids (mg) and usage of opioids on demand (mg) during the first 5 postoperative days for each group. *CPNB*, Continuous peripheral nerve block (group 1); *SSA*, single shot anesthesia (group 2); *GA*, general anesthesia (group 3). Opioid dosages were converted to morphine equivalents. Values indicate median and 5-95 percentile range. Statistics were performed using Mann-Whitney U test.

**Figure 4:**
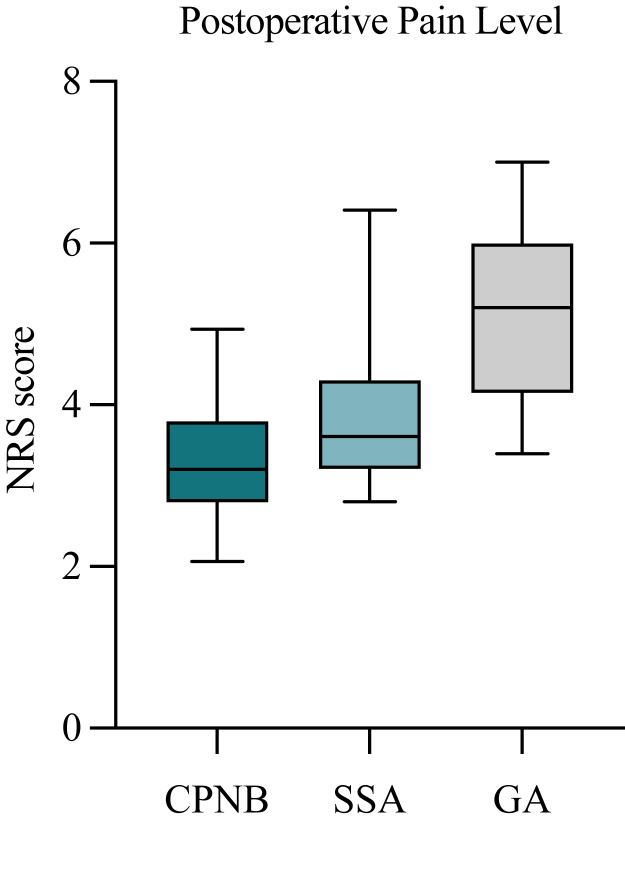
Postoperative pain level. Average NRS scores during the first 5 postoperative days for each group. *NRS*, Numeric Rating Scale; *CPNB*, continuous peripheral nerve block (group 1); *SSA*, single shot anesthesia (group 2); *GA*, general anesthesia (group 3). Values indicate median and 5-95 percentile range. Statistics were performed using Mann-Whitney U test.

Regarding postoperative pain therapy, we found no significant difference in usage of nonopioid pain medication (ibuprofen, *P* = .1569; metamizole, *P* = .8283) (Fig. 2). However, analysis revealed that patients of group 1 received significantly less postoperative opioids (*P* = .0114). Opioid pain medication on demand differed not significantly between group 1 and group 3 (*P* = .6705) (Fig. 3). Moreover, patients in group 1 showed significantly lower average NRS scores during the first 5 postoperative days, indicating substantially better pain management, compared to those in group 3 (*P* < .0001) (Fig. 4).

Comparing patients which received SSA (group 2) and patients who only underwent GA (group 3), patients in group 2 received less intraoperative sufentanil, although with no statistical significance (*P* = .4372). Additionally, there was also no significant difference in intraoperative opioid use between group 1 and group 2 (*P* = .0696) (Fig. 1).

Regarding postoperative pain therapy, we found no significant difference in nonopioid pain medication use (ibuprofen, *P* = .5718; metamizole, *P* = .7383) (Fig. 2). Furthermore, analysis showed that patients of group 2 received less postoperative opioids, although also with no statistical significance (*P* = .1107). Opioid pain medication on demand differed not significantly between group 2 and group 3 (*P* = .5849) (Fig. 3). However, patients in group 2 showed significantly lower NRS scores compared to group 3 (*P* = .0023) (Fig. 4).

Chi-square test with Fisher’s exact test showed no significant difference in distribution of block types (supraclavicular, interscalene, or axillar) between group 1 and group 2 (*P* = .2676) (Table I).

## Discussion

To date, there has been no comparative study investigating the impact of regional anesthesia and a continuous nerve block on perioperative pain management and experienced pain levels during hospitalization following surgery for TEA. We addressed the question of whether an SSA or a CPNB catheter during hospitalization contributes to improved pain scales and reduced opioid consumption.

Our findings reveal significantly lower intraoperative opioid usage in patients who received PNB and nerve block catheter postoperatively (group 1), compared to patients who solely underwent GA (group 3). Furthermore, group 1 used significantly fewer postoperative opioids than group 3. Additionally, patients in group 1 reported significantly lower NRS scores than those in group 3. Similarly, patients who received SSA (group 2) displayed a trend toward reduced intraoperative opioid usage compared to group 3, although this difference did not reach statistical significance. Nevertheless, patients of group 2 reported significantly lower NRS scores than patients in group 3, suggesting better pain control with additional regional anesthesia intraoperatively. Taken together, our results support our hypothesis that the administration of a regional anesthesia, combined with a postoperative nerve block catheter, facilitates improved pain management and reduced opioid consumption. SSA showed no advantage over CPNB, as it did not result in significantly lower intraoperative and postoperative opioid usage.

Total elbow arthroplasty commonly results in significant postoperative pain, which often requires hospitalization for optimal pain management.^18^ Several studies have demonstrated that PNB administered during joint arthroplasty of the lower extremities and postoperative CPNB offer superior pain relief and functional outcomes compared to systemic analgesia, with fewer adverse effects noted.^20^ Previous studies have indicated that PNBs provide effective postoperative anesthesia for various types of shoulder surgeries, including fracture fixation and arthroplasty, with high success rates, relatively low complication rates, and reduced opioid-related side effects.^1^^,^^12^ Interestingly, there is a notable gap in prior research, as no studies have specifically examined the impact of PNB, SSA, and CPNB on postoperative opioid usage and subjective pain levels following surgery for TEA. According to Cardwell et al, perioperative PNBs reduce opioid usage not only postoperatively but also intraoperatively.^5^ This aligns with our study's data on elbow arthroplasty, wherein patients who received PNB exhibited significantly lower intraoperative opioid utilization compared to those who underwent solely GA.

Several studies have demonstrated significant patient benefits of CPNB compared to GA for shoulder sugery.^11^^,^^17^ Borgeat et al showed that administering 0.3% ropivacaine through an interscalene catheter during the first 48 hours after open rotator cuff repair significantly reduced morphine consumption and improved sleep quality on the first postoperative night.^2^ In the present study, we could also show that in patients who received CPNB (group 1), significantly fewer opioids were administered postoperatively, than in patients who solely underwent GA (group 3), indicating more effective pain management in the former group. Additionally, patients receiving only GA (group 3) reported significantly higher NRS scores than those in group 1. As no studies have specifically investigated the impact of PNBs and CPNB in elbow arthroplasty, our study demonstrates that CPNB is an effective technique for achieving adequate postoperative analgesia while reducing opioid consumption in patients undergoing elbow arthroplasty.

In a study by Ilfeld et al, patients who received continuous interscalene block (CISB) for moderately painful arthroscopic shoulder surgery experienced less pain, reduced postoperative opioid consumption, fewer episodes of nausea, and fewer sleep disturbances through the second postoperative day compared to those who received a single injection interscalene block (SISB). It is noteworthy, however, that the SISB group was administered a short-acting local anesthetic (mepivacaine).^16^ In another study by Saliva et al, CISB provided significantly longer analgesia and greater sleep benefits than SISB, even when a long-acting local anesthetic (0.2% ropivacaine) was used for SISB.^23^ In accordance, our analyses revealed that intraoperative SSA did not lead to significantly lower intraoperative and postoperative opioid usage in contrast to patients who received CPNB. Similar findings were reported in a study by Mariano et al, who also used long-acting ropivacaine for outpatient shoulder surgery. They found that CISB provided additional analgesic benefits and reduced opioid consumption, with 67% of patients not requiring supplemental opioids through the second postoperative day. Moreover, CISB resulted in fewer sleep disturbances, higher patient satisfaction, and a longer duration before the first oral analgesic was needed compared to SISB.^19^ Further investigation revealed that following minor arthroscopic shoulder surgery, enhancing a single shot interscalene block with a continuous interscalene ropivacaine infusion significantly reduced pain, particularly during movement, within the first 24 hours.^10^ In the present study, we also used long-acting ropivacaine for SSA as well as for CPNB after TEA. Collectively, the presented studies and our findings highlight that CPNB appears to be significantly superior to SSA in postoperative pain management and opioid reduction. Therefore, the use of a nerve block catheter after TEA may be an effective strategy for optimizing perioperative pain control while also reducing opioid consumption and opioid-related side effects in this patient population.

Cardwell et al demonstrated that the use of perioperative PNBs resulted in lower patient-reported pain scores compared to those who did not receive PNB.^5^ Aligning with our study’s findings, analysis of the present study revealed that patients who received intraoperative SSA, as well as those who were administered CPNB, all reported significantly lower NRS scores during hospitalization. This underscores the enhanced pain relief achieved with regional anesthesia, highlighting its clinical effectiveness in reducing patients’ subjective pain perceptions.

However, a potential complication associated with the use of PNBs is the potential for nerve injuries due to incorrect placement. In a study analyzing peripheral nerve injuries following reverse total shoulder arthroplasty, none of the injuries could be directly attributed to the nerve blocks. The authors suggested that the likely causes of these injuries were traction during arthroplasty and/or significant distalization and lateralization of the implants.^9^ Overall, persistent nerve injuries related to PNBs are uncommon. The incidence of nerve injuries is reported to be 0.01% 6 months after the procedure.^21^ Moreover, it is well known that opioid-related side effects such as vomiting, ileus, dizziness, and respiratory distress may lead to lasting impacts on overall health, independency, and quality of life, which particularly affect elderly patients. Therefore, a reduction of systemic opioid administration by PNB can help avoid aforementioned adverse effects and contribute to better patient outcomes. In summary, we conclude that the overall benefits of PNB, especially of a postoperative nerve block catheter, outweigh the risks of complications. Important to mention at this point is that patients who received elbow arthroplasty and were included in this study were aged on average 70 years. Thus, the investigated patient population and the patients who require elbow arthroplasty in general are mainly elderly individuals, for whom the use of opioids and their significant side effects are even more critical. With the present study, we have demonstrated that the use of nerve block catheters may be an excellent option for elderly patients undergoing surgery for total elbow prosthesis, as they significantly reduce perioperative opioid consumption and, presumably, its severe adverse effects while also improving postoperative subjective pain levels.

This study has some limitations. First, due to its retrospective study design, not all patients received the same type of regional anesthesia as nerve blocks were placed in different positions (interscalene, supraclavicular, or axillar). Furthermore, the dosage of anesthesia varied depending on individual patient characteristics, such as height and weight of the patient, as well as the attending anesthesiologist's discretion. Second, it is important to note that the regional anesthesia was performed by different anesthesiologists at our clinic, potentially contributing to variability in the quality of the nerve block that can never be fully ruled out. However, this variability reflects standard clinical practice conditions. In this regard, we consider this an advantage of our study, as we were still able to demonstrate significant differences in pain relief with CPNB. Third, the sample size of this study was restricted. As a result, a significant effect of SSA on opioid consumption may be observed in larger samples, although its clinical significance remains uncertain. Therefore, our results should be validated in larger cohorts.

To our knowledge, this study is the first to demonstrate that the use of CPNB after TEA is associated with significantly lower intraoperative and postoperative opioid usage in the first 5 days after surgery. Moreover, patient-reported NRS scores were significantly lower in patients who received SSA and CPNB, compared to patients who only underwent GA. Based on our results, we do not consider SSA to provide any additional benefit over CPNB, as SSA did not result in significantly lower intraoperative and postoperative opioid usage. Overall, CPNB appears to be the most effective approach for improving perioperative pain management, reducing opioid consumption and enhancing patient satisfaction regarding postoperative pain levels after surgery for elbow arthroplasty.

## Conclusion

This is the first study on regional anesthesia use in patients undergoing TEA. Overall, our study suggests the use of PNB combined with postoperative nerve block catheter following TEA contributes to reduced perioperative opioid consumption and improved pain levels postoperatively, thereby enhancing patient outcomes and satisfaction during postoperative recovery. Moreover, SSA seems to have no beneficial advantage over CPNB and seems to be more suitable in the context of surgery for elbow arthroplasty, as SSA fails to significantly reduce opioid consumption perioperatively. Especially in an elderly patient population, avoiding systemic opioid usage and its adverse effects may be beneficiary for overall outcomes.

## Disclaimers

Funding: No funding was disclosed by the authors.

Conflicts of interest: The authors, their immediate families, and any research foundation with which they are affiliated have not received any financial payments or other benefits from any commercial entity related to the subject of this article. No financial remuneration the authors, or any member of their family, may have received related to the subject of the article exists for any author.
